# Virologic and Exploratory Immunologic Characterization of Relapsing Human Cytomegalovirus (HCMV) DNAemia in Solid Organ Transplant Recipients: A Prospective Observational Cohort Study

**DOI:** 10.1093/ofid/ofag625

**Published:** 2026-10-01

**Authors:** Golnaz Amidpour, Poramed Winichakoon, Ilona Bahinskaya, Sara Marocco, Marcelo Cypel, Deepali Kumar, Atul Humar, Victor H Ferreira

**Affiliations:** Ajmera Transplant Centre, University Health Network, Toronto, ON, Canada; Institute of Medical Science (IMS), University of Toronto, Toronto, ON, Canada; Ajmera Transplant Centre, University Health Network, Toronto, ON, Canada; Division of Infectious Diseases and Tropical Medicine, Department of Internal Medicine, Faculty of Medicine, Chiang Mai University, Chiang Mai, Thailand; Ajmera Transplant Centre, University Health Network, Toronto, ON, Canada; Ajmera Transplant Centre, University Health Network, Toronto, ON, Canada; Ajmera Transplant Centre, University Health Network, Toronto, ON, Canada; Institute of Medical Science (IMS), University of Toronto, Toronto, ON, Canada; Ajmera Transplant Centre, University Health Network, Toronto, ON, Canada; Institute of Medical Science (IMS), University of Toronto, Toronto, ON, Canada; Ajmera Transplant Centre, University Health Network, Toronto, ON, Canada; Institute of Medical Science (IMS), University of Toronto, Toronto, ON, Canada; Ajmera Transplant Centre, University Health Network, Toronto, ON, Canada; Institute of Medical Science (IMS), University of Toronto, Toronto, ON, Canada; Department of Laboratory Medicine and Pathobiology (LMP), University of Toronto, Toronto, ON, Canada

**Keywords:** DNAemia, HCMV, human cytomegalovirus, organ transplantation, relapse, T cells, viral loads

## Abstract

Relapsing cytomegalovirus DNAemia remains a challenge after organ transplantation. We evaluated longitudinal viral loads and T-cell responses in 62 recipients, of whom 15 relapsed. Week 2 viral loads, but not viral decay slopes or HCMV-specific T-cell responses differed by relapse status, supporting further evaluation of early viral burden.

Solid organ transplant (SOT) recipients are at high risk for serious human cytomegalovirus (HCMV)-related outcomes. HCMV DNAemia, defined as circulating viral DNA exceeding center-specific diagnostic thresholds, and higher viral loads are associated with HCMV disease, impaired allograft outcomes, and immune dysregulation [1–4]. Although antivirals effectively suppress replication, relapse remains common and may prolong treatment, increase toxicity and drug resistance, and raise the risks of HCMV disease, graft dysfunction, and death [1, 5–8]. Risk factors are incompletely defined but include donor-seropositive/recipient-seronegative (D+/R−) serostatus, lung transplantation, absent cell-mediated immunity, lymphopenia, higher presenting viral loads, and failure to clear HCMV after treatment [5, 6, 9–15]. The immune features distinguishing relapse from durable suppression are also unknown. We therefore integrated longitudinal virologic, clinical, and exploratory immunologic data to generate hypotheses for future studies evaluating biomarkers of relapse risk.

## METHODS

### Study Cohort

From November 2013 to September 2018, we prospectively enrolled SOT recipients experiencing their first post-transplant episode of clinically significant HCMV DNAemia at the Ajmera Transplant Centre, UHN, Toronto, Canada. The institutional threshold of ≥1000 IU/mL defined clinically significant DNAemia that prompted antiviral therapy across all SOT types and clinical scenarios. Participants were followed for 6 months after initial virologic suppression for relapse. Supplementary Figure 1 provides the study overview and describes cohort study selection. All participants provided written informed consent, and the UHN Research Ethics Board approved the study.

HCMV prevention strategies are detailed in Supplementary Methods. Plasma viral loads were measured using the clinically validated Cobas CMV qPCR assay (Roche; quantifiable range of 34.5–1.0 × 10^7^ IU/mL). Measurements were analyzed at DNAemia onset, approximately 1 week later and weekly through Week 4. Consistent with published studies [6, 14], relapse was operationally defined as DNAemia ≥1000 IU/mL within 6 months of initial suppression, requiring 2 measurements <200 IU/mL, the institutional threshold, at least 1 week apart.

### Multiparameter Flow Cytometry

Multiparameter flow cytometry was used to assess bulk CD4^+^ and CD8^+^ T-cell phenotypes using a panel of 21 markers, as well as HCMV-specific responses via interferon gamma (IFN-γ) and interleukin-2 (IL-2) intracellular cytokine staining. See Supplementary Methods for full details, including representative gating (Supplementary Figure 2).

### Statistics

Patient-specific viral decay slopes were estimated by ordinary least-squares regression of log_10_ viral load against days from onset of DNAemia, using available measurements and excluding missing values. Eligible participants required a DNAemia onset measurement and at least two additional weekly measurements from Weeks 1 to 4. Undetectable measurements were assigned a value of 17.3 IU/mL, half the assay's lower limit of quantification. The primary analysis included all time points, while an exploratory analysis assessed onset-to-Week 2 kinetics. Slopes were compared by relapse status using two-sided Mann–Whitney *U* tests. T-cell frequencies were similarly compared using Mann–Whitney *U* tests with Benjamini–Hochberg correction. Given this exploratory analysis, *q* < 0.20 identified comparisons of interest. Additional details are provided in the Supplementary Methods.

## RESULTS

### Clinical Characteristics and Demographics of the Cohort

A total of n = 62 SOT recipients were included in the virologic analysis (Table 1). Fifteen (24.2%) relapsed within 6 months of initial suppression. SOT recipients with relapse versus those without relapse had similar age (median [IQR], 60 [47–64] vs 59 [49–65] years), male sex composition (53.3% vs 59.6%), and time from transplantation to DNAemia (240 [75–316] vs 190 [123–305] days). Lung transplantation (73.3% vs 42.6%) and D+/R− serostatus (60.0% vs 40.4%) were more common with relapse. At initial DNAemia, most participants were receiving a calcineurin inhibitor (tacrolimus, 64.5%; cyclosporine, 32.3%), an antimetabolite (mycophenolate, 80.6%; azathioprine, 4.8%), and prednisone (96.8%). Triple maintenance immunosuppression was used by 86.7% who experienced relapse and 78.7% that did not. Median absolute lymphocyte counts at DNAemia were 1.00 (IQR, 0.58–1.31) and 0.70 (IQR, 0.38–1.41), respectively. End-organ HCMV disease occurred in 40.0% versus 21.3%, and HCMV syndrome in 33.3% versus 31.9%. Duration of antiviral treatment at DNAemia was higher in those with relapse with median 66.0 days (IQR, 42.0–88.0) treatment compared to 40 days in those without relapse (IQR, 25.0–58.0). After initial DNAemia suppression, 8/15 (53.3%) versus 7/47 (14.9%) received secondary prophylaxis, while the remaining underwent pre-emptive monitoring.

**Table 1.** Clinical and Demographic Variables of the Cohort

	Relapse, n = 15	No Relapse, n = 47
Age at HCMV DNAemia, in years—median (IQR)	60 (47–64)	59 (49–65)
Male sex—n (%)	8 (53.3)	28 (59.6)
Time from transplantation to DNAemia, in days—median (IQR)	240 (75–316)	190 (123–305)
Type of transplant—n (%)		
Lung	11 (73.3)	20 (42.6)
Non-lung	4 (26.7)	27 (57.4)
Kidney	1 (6.7)	11 (23.4)
Liver	2 (13.3)	10 (21.3)
Heart	0 (0.0)	3 (6.4)
Pancreas	0 (0.0)	1 (2.1)
SKL	1 (6.7)	1 (2.1)
SKP	0 (0.0)	1 (2.1)
Donor/recipient serostatus—n (%)		
D+/R−	9 (60.0)	19 (40.4)
D−/R+	1 (6.7)	10 (21.3)
D+/R+	5 (33.3)	16 (34.0)
Unknown	0 (0.0)	2 (4.3)
Received induction therapy—n (%)	8 (53.3%)	24 (51.1%)
Immunosuppression at the time of DNAemia—n (%)		
Tacrolimus	7 (46.7)	32 (68.1)
Cyclosporine	8 (53.3)	14 (29.8)
Mycophenolate	13 (86.7)	36 (76.6)
Azathioprine	0 (0.0)	4 (8.5)
Prednisone	15 (100)	45 (95.7)
Triple immunosuppression	13 (86.7%)	37 (78.7%)
Absolute lymphocyte count × 10^9^ cells/L—median (IQR)	1.00 (0.58–1.31)	0.70 (0.38–1.41)
CMV disease—n (%)		
End-organ damage	6 (40.0%)	10 (21.3%)
CMV syndrome	5 (33.3%)	15 (31.9%)
Duration of antiviral treatment for initial DNAemia, in days—median (IQR)	66.0 (42.0–88.0)	40.0 (25.0–58.0)
Postsuppression management—n (%)		
Secondary prophylaxis	8 (53.3)	7 (14.9)

Abbreviations: HCMV, human cytomegalovirus; IQR, interquartile range; SKL, simultaneous kidney and liver transplantation; SKP, simultaneous kidney and pancreas transplantation; D+/R−, donor seropositive/recipient seronegative serostatus constellation; D−/R+, donor seronegative/recipient seropositive; D+/R+, donor seropositive/recipient seropositive.

### Viral Load Dynamics in Relation to HCMV Relapse

The median blood viral load at relapse was 2570 IU/mL (IQR: 1390–5760) (Figure 1*A*). Blood viral loads at initial DNAemia were consistently higher among those who subsequently relapsed, reaching statistical significance at 2 Weeks (*P* = .020) (Figure 1*B*, *C*). Using all available measurements from onset to 4 Weeks, median viral decay slopes were more negative—suggestive of faster viral load control—among participants without relapse compared to those with relapse (−0.074 versus −0.052 log_10_ IU/mL/day), although the difference was not statistically significant (*P* = .29). In an exploratory analysis restricted to early viral load kinetics (onset-to-2 Weeks), greater separation was observed between the no relapse and relapse groups (−0.054 versus −0.023 log10 IU/mL/day), but this difference also did not reach statistical significance (*P* = .077) (Figure 1*D*). Viral decay slopes did not differ between D+/R− and R+ participants (Figure 1*E*).

**Figure 1.**
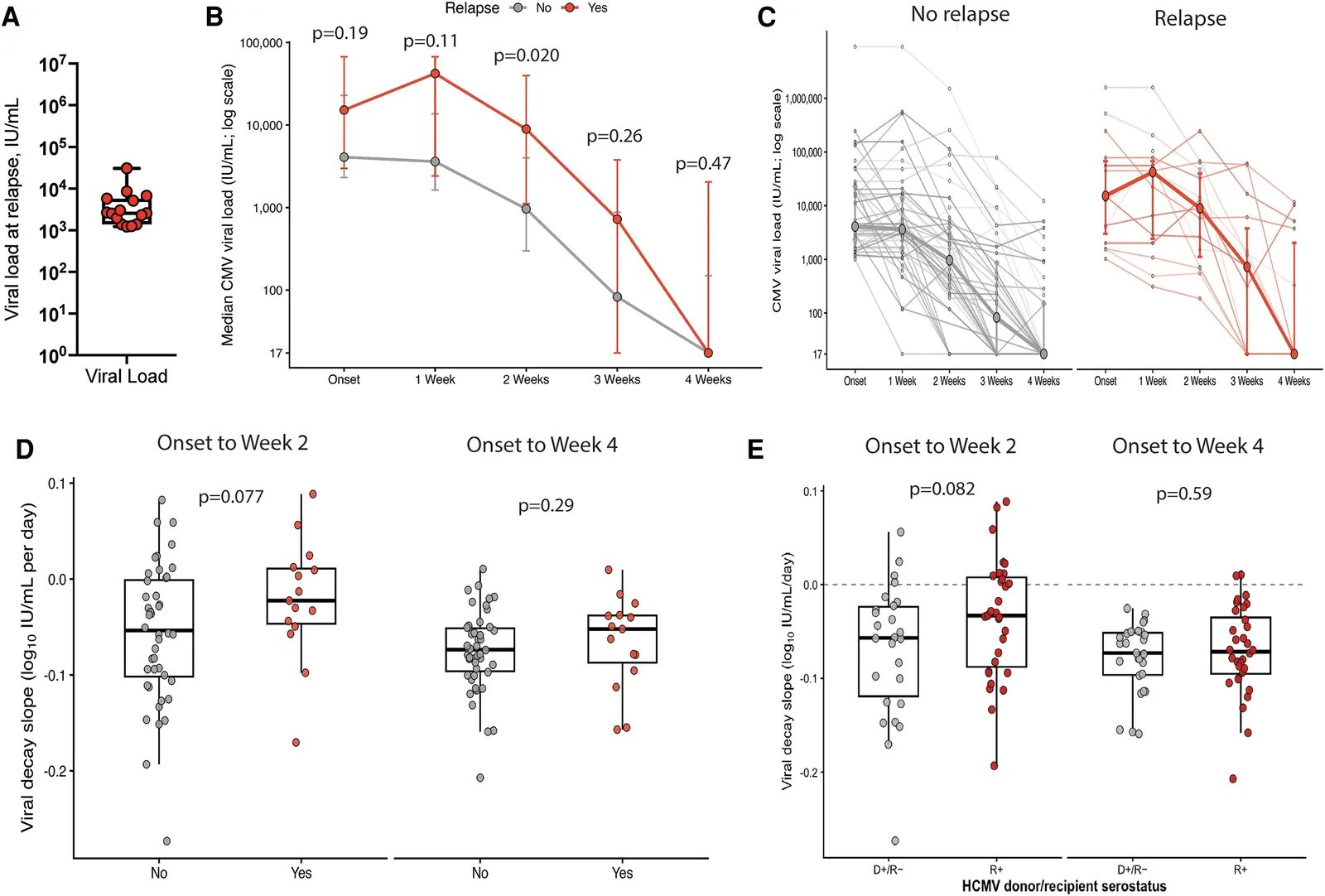
Viral load measurements and relation to HCMV relapse. *A*, Viral load at the time of relapse. *B*, Clinical blood viral loads in international units per milliliter (IU/mL) across visits in solid organ transplant recipients with and without human cytomegalovirus (HCMV) relapse. Horizontal lines indicate the median and the vertical line indicates the interquartile range. Each dot is one patient. *C*, Individual viral load trajectories, where each line represents one patient. *D*, Viral decay slopes in log_10_-transformed IU/mL for patients with and without HCMV relapse, and (*E*) according to donor-recipient HCMV serostatus. *P*-values indicate the Mann–Whitney *U* test result for the indicated comparisons.

### Exploratory T-Cell Correlates of HCMV Relapse

Exploratory T-cell phenotyping was performed in a subset of SOT recipients (n = 33), of which 27.3% developed relapse (Supplementary Table 1). HCMV-specific CD4^+^ (Supplementary Figure 3A–C) and CD8^+^ (Supplementary Figure 3D–F) T-cell responses were found in subsets of participants and primarily consisted of IL-2 monofunctional and IFN-γ monofunctional T cells. HCMV-specific polyfunctional CD8^+^ T cells were rarely detected, while polyfunctional CD4^+^ T-cell responses were not detected. HCMV-specific T-cell responses were similar between those with and without relapse. Bulk CD3^+^, CD4^+^, and CD8^+^ T-cell frequencies were also similar between those with and without relapse (Supplementary Figure 3G–I).

Phenotypic and functional marker expression in bulk CD4^+^ and CD8^+^ T cells were compared between participants with and without HCMV relapse. At 1 Week post-DNAemia, we identified higher frequencies of CTLA-4^+^ CD4^+^ (*P* = .0056, *q* = 0.12) and CTLA-4^+^ CD8^+^ (*P* = .0012, *q* = 0.025) T cells in those experiencing relapse (Supplementary Figure 4). At 4 Weeks, a similar pattern was observed, with higher frequencies of CTLA-4 expressing cells associated with relapse in both T-cell compartments (CTLA4^+^ CD4^+^: *P* = .0076, *q* = 0.16; CTLA4^+^ CD8^+^: *P* = .0092, *q* = 0.19). No other T-cell markers were associated with relapse status after multiplicity correction (Supplementary Table 2).

## DISCUSSION

In this study, we integrated virologic, clinical and exploratory immunologic profiling to identify correlates of relapsing HCMV DNAemia after initial virologic suppression in a cohort of SOT recipients. Approximately, a quarter (24.2%) of the overall cohort experienced relapse. This is similar to rates published in earlier studies, where ∼20–30% of SOT recipients experienced relapse [6–9, 11, 16, 17].

Early plasma HCMV loads, particularly at Week 2 after DNAemia, were significantly higher among participants who subsequently relapsed, whereas later viral loads and decay slopes did not differ. Thus, early absolute viral burden may be more informative than clearance rate. Previous studies have similarly associated relapse with higher presenting viral loads, treatment-phase kinetics, and failure to clear HCMV by treatment completion [1, 5, 6, 9, 11, 13]. Early post-DNAemia viral loads may therefore provide actionable prognostic information for relapse risk.

Lung transplant recipients were overrepresented among participants who relapsed, which may reflect their greater net state of immunosuppression or other organ-specific factors [18–25], though rates of triple immunosuppression were similar between those with and without relapse in our study. The limited number of relapse events precluded reliable separation of the effects of transplant type and immunosuppressive burden; therefore, residual confounding cannot be excluded. Although the association with lung transplantation was not statistically significant in our cohort, its consistency with prior observations [6, 15] warrants further investigation in larger studies.

Our study has several important limitations. The modest number of relapse events limited power to identify independent predictors, particularly in immunologic analyses. Findings should therefore be considered exploratory and hypothesis-generating. HCMV prevention strategies also varied, reflecting real-world use of prophylaxis or pre-emptive approaches according to serostatus, organ type, and immunosuppression.

Polyfunctional HCMV-specific T-cell responses were infrequently detected by intracellular cytokine staining after peptide stimulation, potentially reflecting biological and technical factors. Active DNAemia, lymphopenia, and triple immunosuppression may have impaired detection of highly functional subsets. Although background subtraction quantified peptide-inducible cytokine production above unstimulated controls, it may not capture the total HCMV-associated response. During active DNAemia, spontaneous cytokine expression may represent meaningful in vivo activation rather than technical background. Thus, similar background-subtracted responses do not exclude broader differences in T-cell activation between outcome groups. Our study was also limited in that we could not perform antigen-specific T-cell phenotyping due to low cell counts. Larger studies incorporating HCMV-specific analyses, non-DNAemia controls, longitudinal immunosuppressive exposures, donor-recipient serostatus and adequately powered multivariable models are needed to determine whether immune markers independently associate with relapse or reflect broader post-transplant immunosuppression.

Taken together, these findings suggest that relapse after HCMV suppression may reflect early viral burden and transplant-specific clinical factors, while the immunologic findings remain exploratory. Validation in larger, independent cohorts is required to determine whether early viral loads improve risk stratification. Better defining relapse risk may ultimately support more individualized monitoring and antiviral management for SOT recipients with HCMV.

## Supplementary Material

ofag625_Supplementary_Data

## Supplementary Data

Supplementary materials are available at *Open Forum Infectious Diseases* online. Consisting of data provided by the authors to benefit the reader, the posted materials are not copyedited and are the sole responsibility of the authors, so questions or comments should be addressed to the corresponding author.
