# Supplementary material for: Virologic and Exploratory Immunologic Characterization of Relapsing Human Cytomegalovirus (HCMV) DNAemia in Solid Organ Transplant Recipients: A Prospective Observational Cohort Study
Source: Open Forum Infect Dis. 2026 Oct 1;13(10):ofag625. doi: 10.1093/ofid/ofag625 (PMC13660685; doi:10.1093/ofid/ofag625)
Supplement: Supplementary file 1 [file ofag625_supplementary_data.pdf]

Supplementary Data

Table of Contents

Pages

Supplementary Methods……………………………………………………………………2-5

Supplementary Figures

Supplementary Figure 1 - Study outline**.** ………………………………………………6

Supplementary Figure 2 - Overall gating strategies and representative gating……....7-9

Supplementary Figure 3 - Antigen-specific and global T cell frequencies in those with and without relapse………………………………………………...……………...10-11

Supplementary Figure 4 - T cell marker diversity in SOT recipients with human cytomegalovirus DNAemia according to relapse status……………………….………..12-13

Supplementary Tables

Supplementary Table 1 - Demographics and clinical characteristics of cellular cohort. ……………………………………………………………………………………..14-15

Supplementary Table 2 - Bulk T cell marker expression according to relapse status. ……………………………………………………………………………………..16-22

Supplementary References…………………………………………………………………23

Supplementary Methods

HCMV Prevention Strategies

At University Health Network (UHN), human cytomegalovirus (HCMV) prevention strategies are tailored according to transplanted organ, donor-recipient HCMV serostatus, and induction immunosuppression. Lung transplant recipients who are HCMV-seropositive (R+) receive six months of antiviral prophylaxis, whereas HCMV donor-seropositive/recipient-seronegative (D+/R−) lung recipients receive nine months. D+/R− recipients of all other organs receive six months of prophylaxis. Among R+ recipients, heart and kidney-pancreas recipients receive three months of prophylaxis, as do kidney recipients treated with thymoglobulin induction. In contrast, R+ liver recipients and R+ kidney recipients who do not receive thymoglobulin induction are managed using a pre-emptive monitoring strategy.

Multiparameter Flow Cytometry

Peripheral blood mononuclear cells (PBMCs) were isolated from peripheral blood by Ficoll density-gradient centrifugation and cryopreserved in freezing medium containing fetal bovine serum (FBS) and dimethyl sulfoxide (DMSO). For immunologic analyses, cells were rapidly thawed in a 37°C water bath and added dropwise to prewarmed Roswell Park Memorial Institute (RPMI) medium supplemented with 10% FBS, L-glutamine, and antibiotics. Following gentle centrifugation to remove residual DMSO, cells were resuspended in complete RPMI medium and rested at 37°C for two hours before peptide stimulation. PBMC were incubated overnight with a pool of 99 HCMV peptides consisting of defined class I and II-restricted T cell epitopes (PepMix™ Pan-CMV Select, JPT Peptide Technologies). Each antigen was used at a

concentration of 5ug/mL. After the first hour of incubation with peptides, a protein transport inhibitor (ThermoFisher) was also added to the cells to prevent cytokine release. Media containing an equivalent DMSO percentage as peptide-stimulated samples was used as a negative control, and PBMC stimulated with eBioscience™ Cell Stimulation Cocktail (ThermoFisher) consisting of phorbol 12-myristate 13-acetate (PMA) and ionomycin, was used as a positive control.

After overnight incubation, cells were prepared for flow cytometry. After viability staining (FVS 575V) and Fc blocking, PBMC were incubated with a cocktail of antibodies against surface markers, including anti-human CD3-BV480, CD4-BUV805, CD8-BUV496, CD223/LAG-3-BV786, CD366/TIM-3-PE-CF594, CD160-AF647, CD279/PD-1-BUV661, TIGIT-BUV615, CD357/GITR-BV650, CD28-RB780, KLRG1-BB700, CD57-BB515, CD154/CD40L-PECy5, CD69-APCH7, CD95/Fas-BUV737, CD134/OX40-BUV563, CD137/4-1BB-BUV395 and CTLA-4/CD152-PE-Cy7. After fixation and permeabilization, cells were incubated with an intracellular antibody cocktail consisting of anti-human Ki67-BV605, TCF-1-PE, interferon gamma (IFN-γ)-BV750, interleukin 10 (IL-10)-BV711, granzyme B (GzmB)-BV421, and IL-2-R718. Antibody staining was performed in the presence of BD Horizon™ Brilliant Stain Buffer to minimize staining artefacts. All antibodies and flow reagents were purchased from BD Biosciences. Data was captured on a BD Symphony A3 instrument in the SickKids-UHN Flow Cytometry Facility, acquired using BD FacsDiva version 6.1.3, and analyzed using FlowJo version 10.10.

Antigen-specific T cells were defined in terms of interferon gamma (IFN-γ) and interleukin-2 (IL-2) expression in peptide-stimulated T cells. Monofunctional cells included IFN-γ-monofunctional ($IFN\text{-}\gamma^{+}IL\text{-}2^{-}$) and IL-2 monofunctional ($IFN\text{-}\gamma^{-}IL\text{-}2^{+}$) T cells, and

polyfunctional T cells were defined as those expressing both IFN-γ and IL-2. A minimal T cell frequency of 0.01%, after subtraction of background cytokine expression was set as the limit of quantitation, as per prior studies[1,2]. Following subtraction of cytokine expression in the unstimulated condition, HCMV-specific responses below the lower limit of quantification, including responses with no detectable events, were assigned a value of 0.005% (half the limit of quantification). Samples were considered evaluable if the positive control generated an IFN-γ^+IL-2^+ $CD4^+$ or $CD8^+$ T cell response exceeding the corresponding background response by at least three standard deviations. Two samples failed these criteria and were excluded from the antigen-specific analysis.

Statistical Analyses

Descriptive statistics were used to summarize demographic and clinical data. Categorical data were analyzed using Chi-square or Fisher's Exact test, as indicated by sample size, while continuous data was analyzed using Mann-Whitney U testing. Statistical analyses and figures were generated using GraphPad Prism version 10.6, R version 4.6.0 and RStudio version 2026.6.0.242.

To assess within-patient viral clearance kinetics, for each participant with a PCR test result at DNAemia, and at least two additional measurable viral load test results from 1 Week to 4 Weeks post-DNAemia, a per-patient viral decay slope was calculated by fitting a simple linear regression model to $\log_{10}$-transformed viral loads across all available timepoints, with time modeled as days from the onset of DNAemia. Regressions were fitted separately for each participant using ordinary least squares, using all available viral load measurements and omitting missing values. To permit $\log_{10}$ transformation, undetectable viral-load measurements were

assigned a fixed value of 17.3 IU/mL, approximately half the assay's lower limit of quantification of 34.5 IU/mL. This substitution was used only for calculation of individual viral decay slopes. The primary viral clearance analysis used all available measurements from DNAemia onset to 4 Weeks post-onset. Because early treatment response could be more informative than the subsequent decline toward the assay detection limit, an exploratory analysis calculated separate patient-specific slopes using DNAemia to Week 2 measurements. Slopes were compared between relapse groups using two-sided Mann-Whitney U test.

For the multiparameter T cell analysis, T cell subset frequencies were compared between those with or without relapse using Mann-Whitney U test, with multiple comparison correction performed using the Benjamini-Hochberg method. A threshold of $q<0.2$ was chosen to balance sensitivity and specificity in this exploratory, high-dimensional analysis. Use of liberal false discovery rate (FDR) thresholds, such as 20%, has been applied in other high-throughput contexts where discovery of candidate features is the primary goal[3].

Supplementary Figure 1

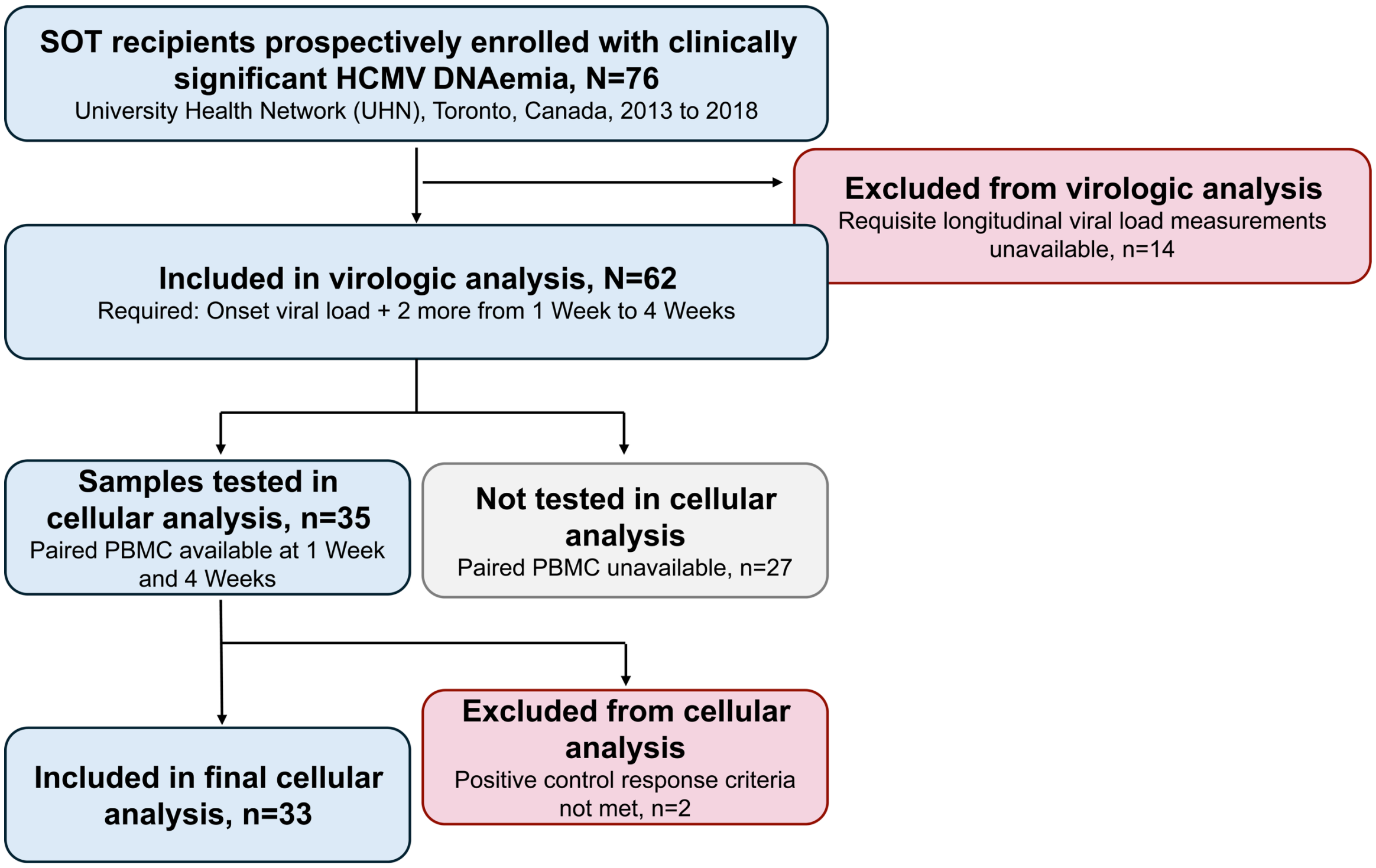

Supplementary Figure 1 – Study outline. This prospective observational study enrolled 76 solid organ transplant (SOT) recipients with their first episode of clinically significant human cytomegalovirus (HCMV) DNAemia at the University Health Network in Toronto, Ontario, Canada between November 2013 and September 2018. Fourteen participants lacked the required longitudinal viral load measurements for virologic assessment and were excluded, leaving 62 with a viral load at onset and at least two additional measurements between Weeks 1 and 4 for virologic analysis. Participants were followed for six months after viral suppression to identify relapse. Paired Week 1 and Week 4 peripheral blood mononuclear cells (PBMC) samples were available from 35 participants; two failed positive-control criteria, leaving 33 for exploratory T cell analyses.

Supplementary Figure 2

A

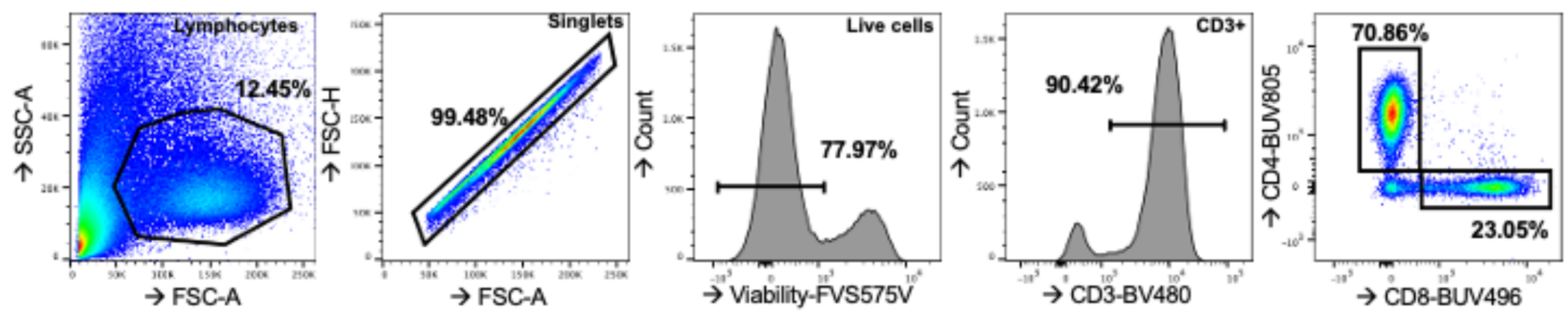

B

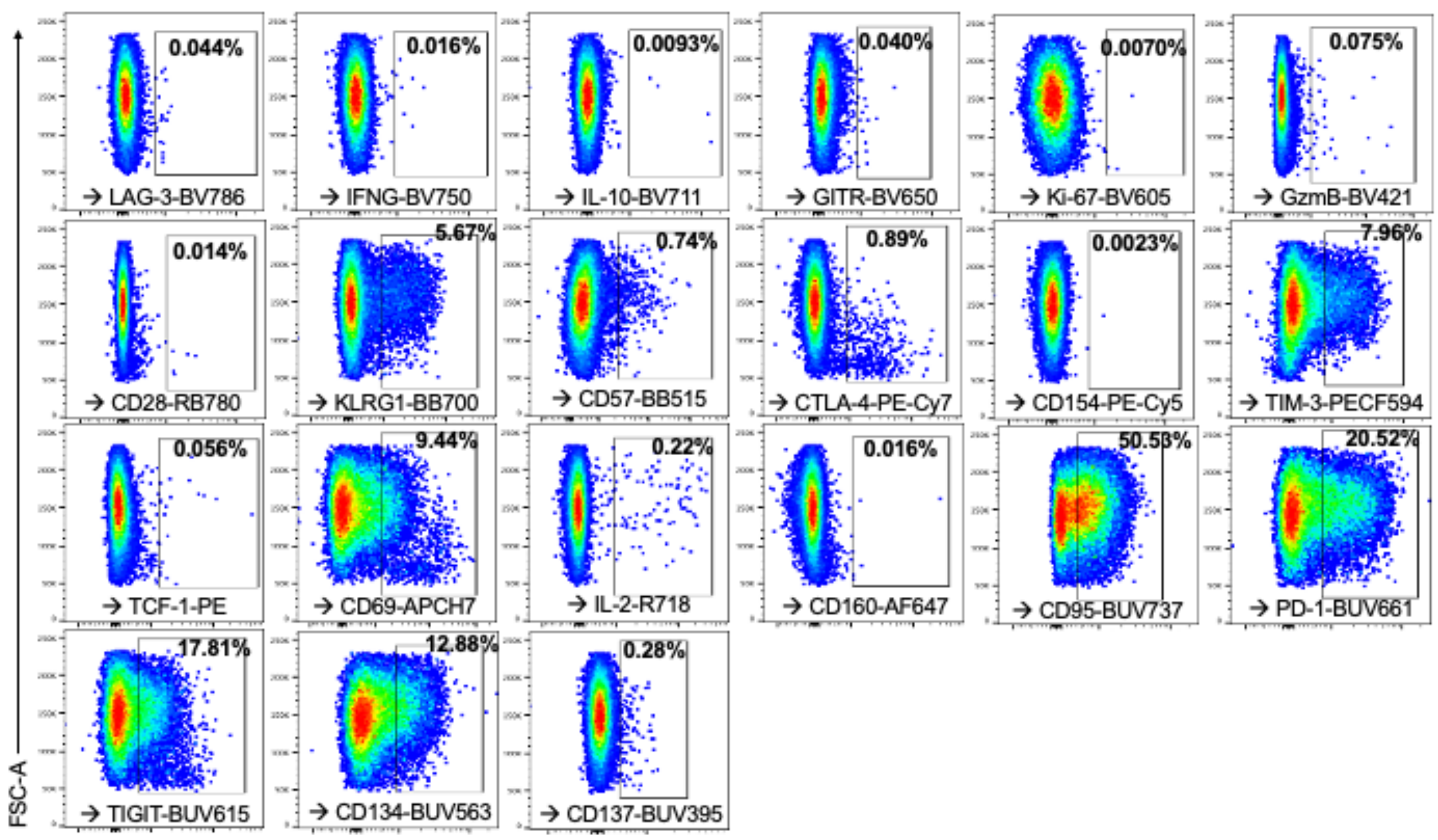

C

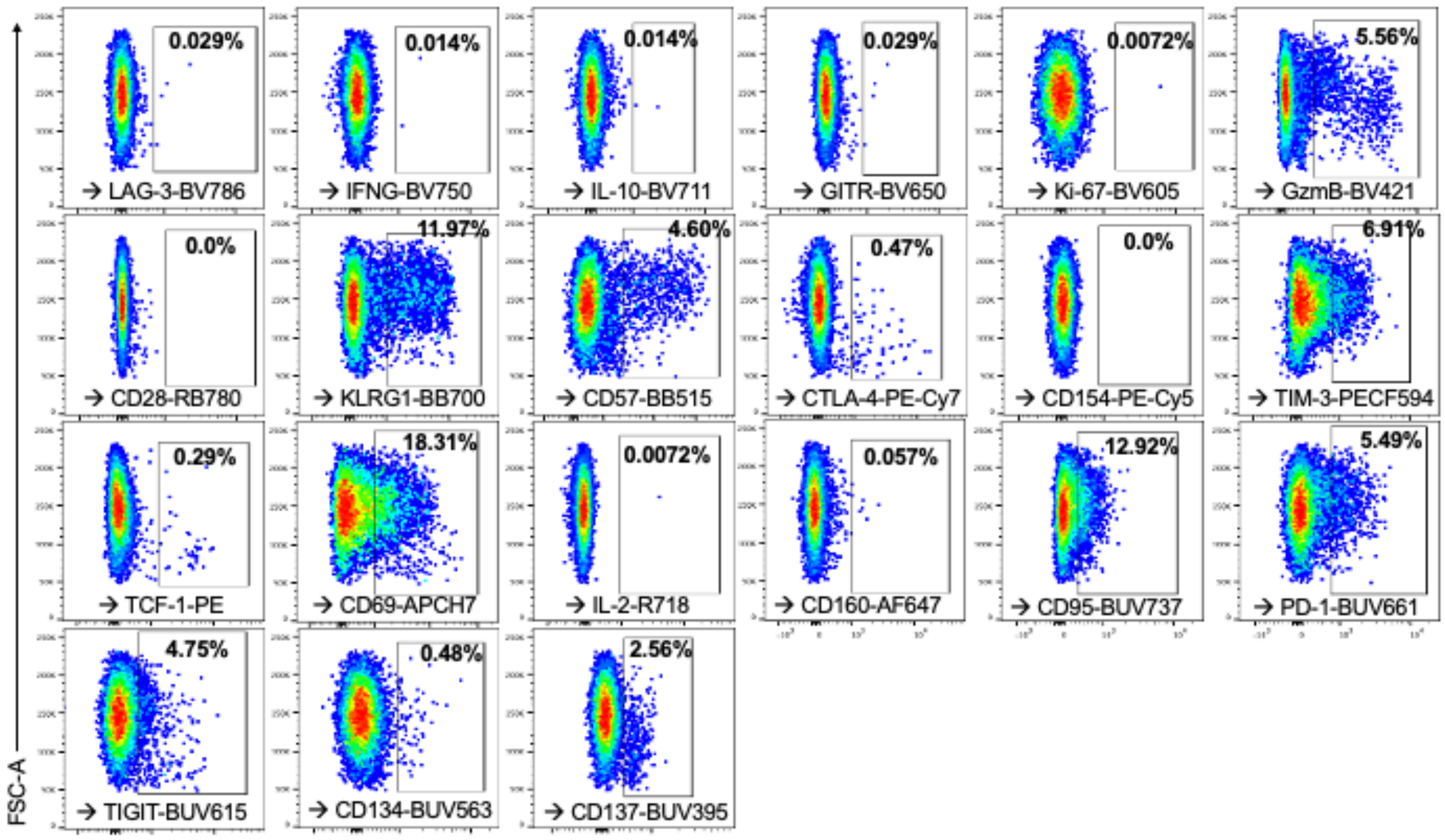

0.029%
LAG-3-BV786
0.014%
IFNG-BV750
0.014%
IL-10-BV711
0.029%
GITR-BV650
0.0072%
Ki-67-BV605
5.56%
GzmB-BV421
0.0%
CD28-RB780
11.97%
KLRG1-BB700
4.60%
CD57-BB515
0.47%
CTLA-4-PE-Cy7
0.0%
CD154-PE-Cy5
6.91%
TIM-3-PECF594
0.29%
TCF-1-PE
18.31%
CD69-APCH7
0.0072%
IL-2-R718
0.057%
CD160-AF647
12.92%
CD95-BUV737
5.49%
PD-1-BUV661
4.75%
TIGIT-BUV615
0.48%
CD134-BUV563
2.56%
CD137-BUV395
FSC-A

D

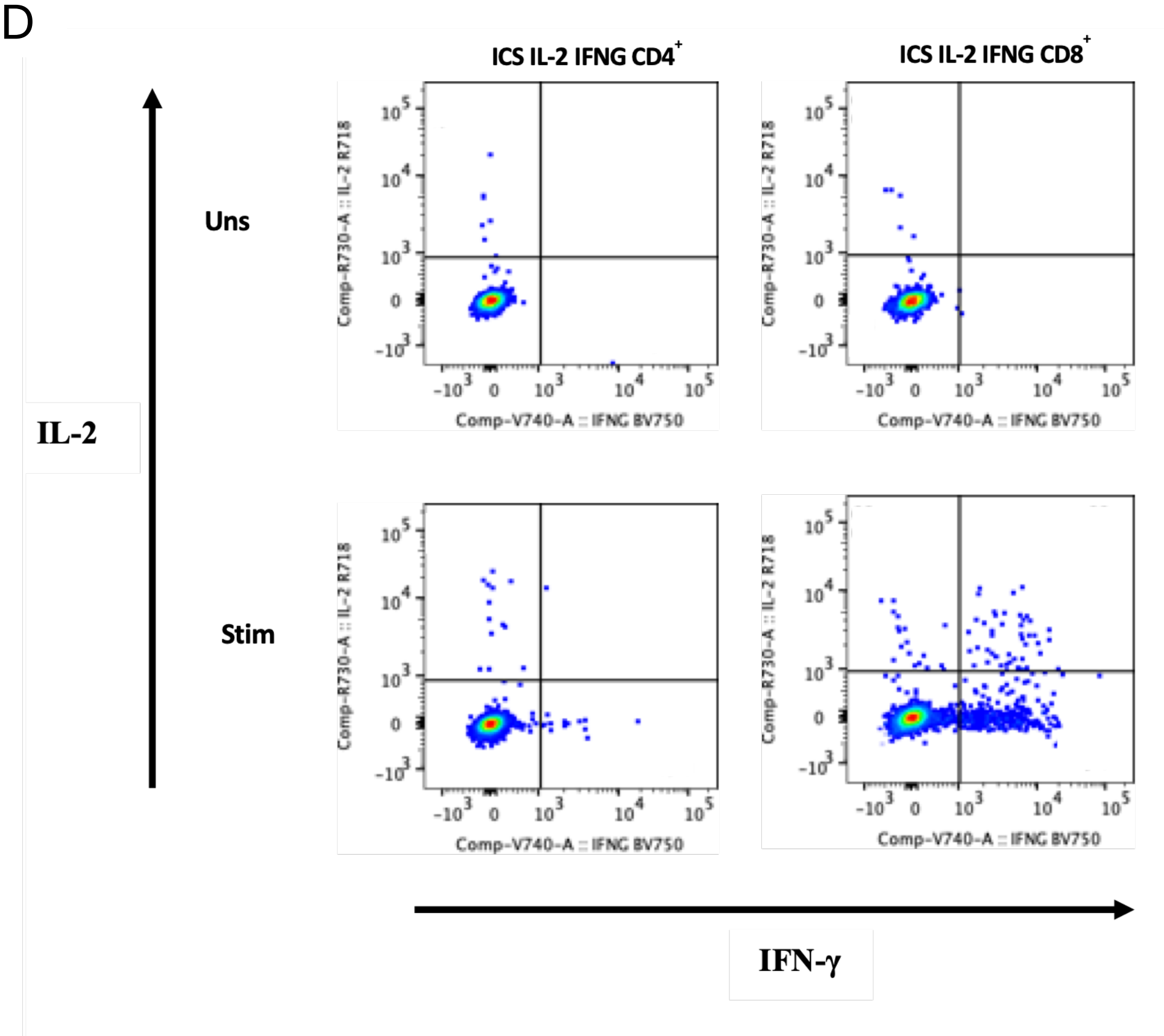

ICS IL-2 IFNG CD4+
ICS IL-2 IFNG CD8+
Uns
Stim
IL-2
IFN-γ
Comp-R730-A :: IL-2 R718
Comp-V740-A :: IFNG BV750

Supplementary Figure 2 – Overall gating strategies and representative gating. (A) Pre-gating facilitated the identification of live, singlet lymphocytes using forward and side scatter channels. Arrows indicate the sequence of hierarchical gates. Number in each plot refers to the frequency of the gated subset. Representative marker gating on bulk (B) $CD4^+$ T cells and (C) $CD8^+$ T cells. (D) A representative HCMV-specific T cell response using intracellular cytokine staining from one participant following overnight peptide stimulation. Control cells not receiving peptide stimulation were used to determine the frequency of background cytokine expression. Abbreviations: FSC – forward scatter, SSC – side scatter, CLTA-4 - cytotoxic T lymphocyte-associated protein 4, GITR - glucocorticoid-induced tumor necrosis factor receptor-related protein, IFNG/IFN-γ – interferon gamma, IL-2 – interleukin 2 – interferon gamma, GzmB – granzyme B, IL - interleukin, KLRG1 - killer cell lectin-like receptor subfamily G member 1, LAG-3 - lymphocyte activation gene 3, PD-1 - programmed cell death protein 1, TCF-1 - T cell factor 1, TIGIT - T cell immunoreceptor with Ig and ITIM domains, TIM-3 - T cell immunoglobulin and mucin domain-containing protein 3, uns – unstimulated, stim – stimulated.

Supplementary Figure 3

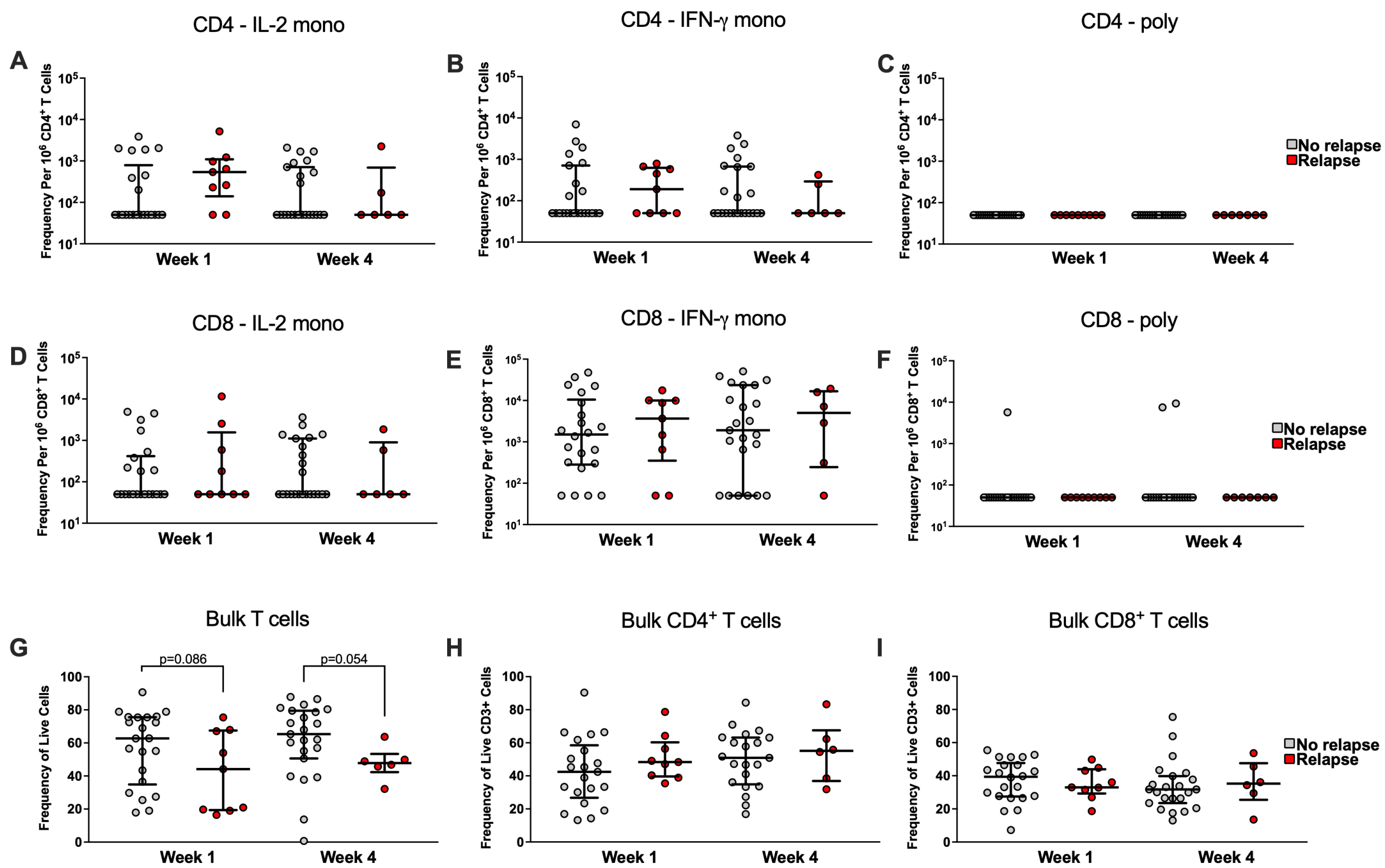

Supplementary Figure 3 - Antigen-specific and global T cell frequencies in those with and without relapse. Frequencies of human cytomegalovirus (HCMV)-specific $CD4^+$ (A-C), and $CD8^+$ (D-F) T cells, and global/bulk T cell frequencies (G-I) in transplant recipients with or without relapse of DNAemia. Testing was performed at one-week (V1) and four-weeks (V4) post-DNAemia. Each dot represents one patient. HCMV-specific T cells are shown in terms of monofunctional interleukin 2 (IL-2; A, D), monofunctional interferon gamma (IFN-γ; B, E), and polyfunctional (IFN-γ+IL-2+: C, F) T cells. Antigen-specific T cell frequencies are reported in terms of the parental subset population. For global T cell frequencies, $CD3^+$ cell frequencies were reported in terms of live lymphocytes, and total $CD4^+$ and $CD8^+$ T cell frequencies are reported in terms of $CD3^+$ cells. Horizontal lines indicate the median with the vertical lines

indicating the interquartile range. P-values indicate the Mann-Whitney U test result for the indicated comparisons.

Supplementary Figure 4

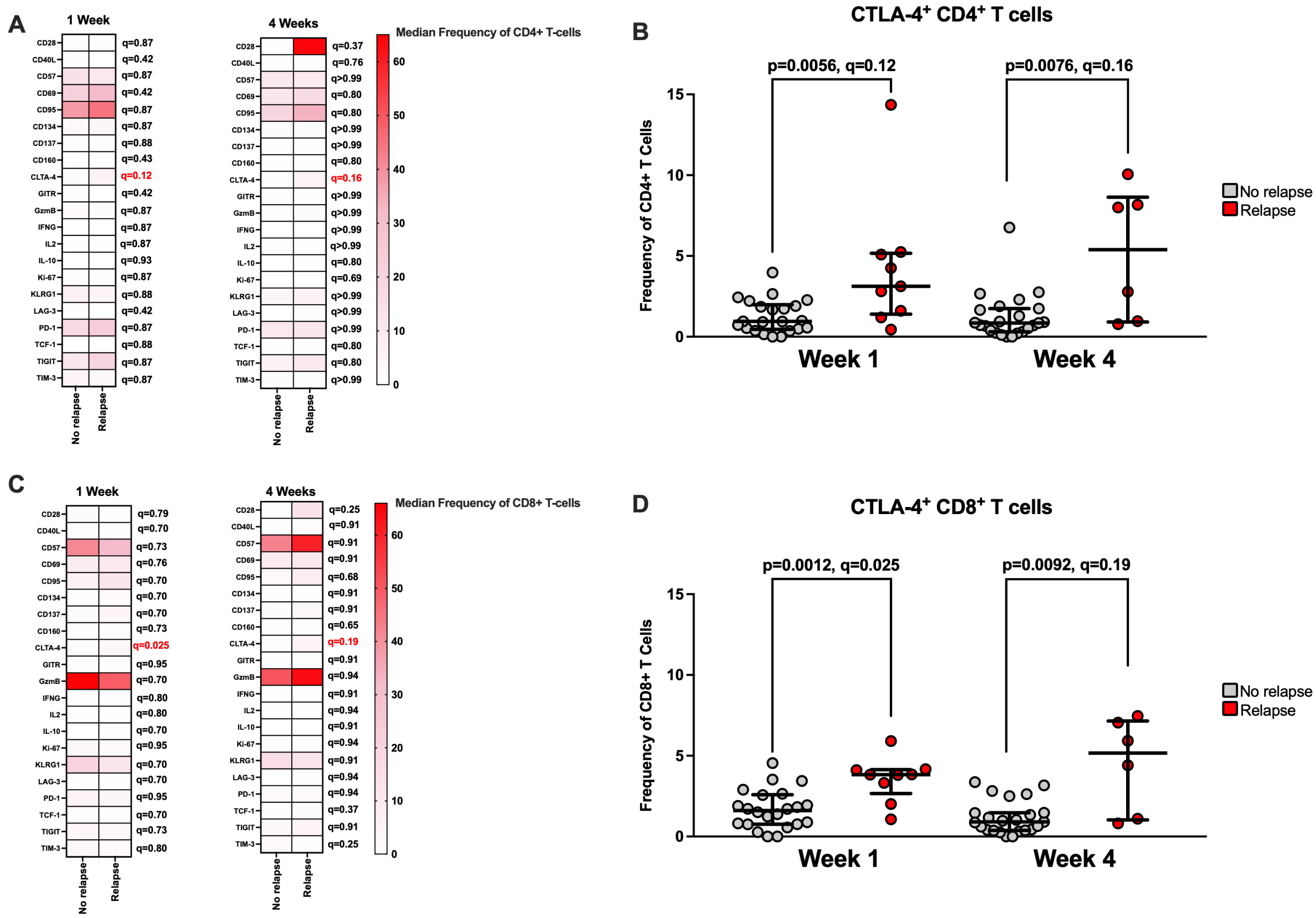

Supplementary Figure 4 - T cell marker diversity in SOT recipients with human cytomegalovirus DNAemia according to relapse status. Heatmaps displaying the median frequency of bulk (A) $CD4^+$ and (C) $CD8^+$ T cells baring the different markers listed at one-week (V1) and four-weeks (V4) post-DNAemia according to relapse status. The colour bar represents the frequency (%) of the indicated marker expressed in terms of the parental $CD4^+$ or $CD8^+$ T cell population. Cytotoxic T-lymphocyte-associated protein 4 (CTLA-4)-expressing $CD4^+$ (B) and $CD8^+$ (D) T cell frequencies at visit 1 (V1) and V4 in patients with or without relapse. Horizontal lines indicate the median and the vertical line indicates the interquartile range. Each

dot is one patient. Statistical testing was performed via Mann-Whitney U test between the indicated comparisons, with adjusted q-values via the Benjamini-Hochberg method. Abbreviations: CLTA-4 - cytotoxic T lymphocyte-associated protein 4, GITR - glucocorticoid-induced tumor necrosis factor receptor-related protein, IFNG – interferon gamma, GzmB – granzyme B, IL - interleukin, KLRG1 - killer cell lectin-like receptor subfamily G member 1, LAG-3 - lymphocyte activation gene 3, PD-1 - programmed cell death protein 1, TCF-1 - T cell factor 1, TIGIT - T cell immunoreceptor with Ig and ITIM domains, TIM-3 - T cell immunoglobulin and mucin domain-containing protein 3.

Supplementary Table 1 – Demographics and clinical characteristics of cellular cohort.

**Characteristic**	Overall T cell cohort (n=33)
**Age, in years - median (IQR)**	58.0 (46.5-66.0)
**Male Sex – n (%)**	20 (60.6%)
**Time from transplant to DNAemia, in days – median (IQR)**	259 (130-327)
**Transplant Type – n (%)**	
**Kidney**	5 (15.2%)
**Liver**	4 (12.1%)
**Lung**	20 (60.6%)
**Heart**	2 (6.1%)
**Kidney and Pancreas**	1 (3.0%)
**Liver and Kidney**	1 (3.0%)
**HCMV Serostatus – n (%)**	
**D+/R+**	9 (27.3%)
**D–/R+**	4 (12.1%)
**D+/R–**	20 (60.6%)
**Immunosuppression at DNAemia – n (%)**	
**Tacrolimus**	19 (57.6%)
**Cyclosporine**	14 (42.4%)
**Mycophenolate**	28 (84.8%)
**Azathioprine**	2 (6.1%)
**Prednisone**	32 (97.0%)
**Triple immunosuppression**	29 (87.9%)
**Absolute lymphocyte count x 10^9 cells/L – median (IQR)**	0.94 (0.56-1.57)
**HCMV disease – n (%)**	
**End-organ damage**	7 (21.2%)
**CMV syndrome**	14 (42.4%)

**Duration of antiviral treatment for initial DNAemia, in days – median (IQR)**	48.0 (33.0-70.5)
**Post-suppression management – n (%)**	
**Secondary prophylaxis**	16 (48.5)

Abbreviations: IQR - interquartile range, D+/R– - donor seropositive/recipient seronegative serostatus constellation, D–/R+ - donor seronegative/recipient seropositive, D+/R+ - donor seropositive/recipient seropositive.

Supplementary Table 2 – Bulk T cell marker expression according to relapse status.

CD4⁺ T cells								
	**V1**				**V4**			
	**No relapse, median (IQR)**	**Relapse, median (IQR)**	**p-value**	**q-value**	**No relapse, median (IQR)**	**Relapse, median (IQR)**	**p-value**	**q-value**
**CD28**	0.28 (0.081-62.52)	0.35 (0.13-68.64)	0.56	0.87	0.27 (0.11-61.03)	64.18 (1.84-86.55)	0.036	0.37
**CD40L**	0.024 (0.0-0.15)	0.0 (0.0-0.003)	0.065	0.42	0.0070 (0.0-0.10)	0.081 (0.006-0.59)	0.15	0.76
**CD57**	7.90 (5.07-15.94)	6.05 (5.02-15.70)	0.62	0.87	10.94 (5.73-18.10)	9.82 (5.20-13.31)	0.61	>0.99
**CD69**	12.71 (6.14-18.77)	18.18 (13.19-24.23)	0.086	0.42	12.37 (6.98-17.84)	16.56 (9.15-21.79)	0.32	0.80
**CD95**	26.36 (15.03-35.84)	36.29 (16.35-44.07)	0.43	0.87	19.96 (7.04-32.23)	33.72 (15.08-38.15)	0.23	0.80

**CD134**	1.84 (0.62-4.56)	2.22 (1.30-5.10)	0.60	0.87	1.74 (0.78-3.50)	1.75 (0.18-5.02)	0.81	>0.99
**CD137**	0.10 (0.023-0.39)	0.13 (0.0-0.33)	0.75	0.88	0.13 (0.024-0.44)	0.12 (0.055-0.95)	0.77	>0.99
**CD160**	0.077 (0.0-0.15)	0.16 (0.047-0.71)	0.12	0.43	0.089 (0.014-0.20)	0.29 (0.012-0.63)	0.33	0.80
**CTLA-4**	0.95 (0.46-1.98)	3.12 (1.40-5.16)	0.0056	0.12	0.85 (0.31-1.74)	5.39 (0.92-8.64)	0.0076	0.16
**GITR**	0.10 (0.0-0.55)	0.0 (0.0-0.057)	0.058	0.42	0.022 (0.0-0.15)	0.052 (0.0-0.19)	0.72	>0.99
**GzmB**	1.21 (0.57-4.89)	0.85 (0.29-3.49)	0.47	0.87	0.98 (0.19-10.80)	1.64 (0.38-4.68)	0.94	>0.99
**IFN-γ**	0.0 (0.0-0.033)	0.0 (0.0-0.030)	0.68	0.87	0.0 (0.0-0.071)	0.0 (0.0-0.41)	>0.99	>0.99

**IL-2**	0.058 (0.0-0.14)	0.028 (0.0-0.29)	0.71	0.87	0.024 (0.0-0.15)	0.049 (0.0-0.15)	0.92	>0.99
**IL-10**	0.0 (0.0-0.035)	0.0 (0.0-0.10)	0.93	0.93	0.0 (0.0-0.0)	0.0041 (0.0-0.31)	0.23	0.80
**Ki-67**	0.61 (0.026-1.22)	0.42 (0.21-0.72)	0.68	0.87	0.088 (0.0-0.47)	0.47 (0.098-1.31)	0.099	0.69
**KLRG1**	3.75 (1.65-7.35)	2.97 (0.82-9.37)	0.84	0.88	4.29 (1.88-7.98)	5.61 (0.33-13.24)	0.94	>0.99
**LAG-3**	0.036 (0.0-0.18)	0.0 (0.0-0.024)	0.10	0.42	0.0 (0.0-0.089)	0.016 (0.0-0.13)	0.84	>0.99
**PD-1**	9.64 (5.32-19.76)	13.31 (6.88-17.87)	0.68	0.87	10.56 (5.41-20.00)	11.77 (7.93-14.62)	>0.99	>0.99
**TCF-1**	0.086 (0.0-0.46)	0.13 (0.020-0.18)	0.81	0.88	0.17 (0.0-0.42)	0.012 (0.0-0.28)	0.35	0.80

**TIGIT**	6.41 (3.54-14.04)	11.29 (5.66-12.66)	0.45	0.87	6.57 (3.91-10.29)	10.48 (4.91-11.29)	0.40	0.80
**TIM-3**	2.47 (0.14-3.45)	1.70 (0.094-2.55)	0.37	0.87	1.68 (0.12-3.03)	0.72 (0.35-2.11)	0.78	>0.99
**$CD8^+$ T cells**								
	**V1**				**V4**			
	**No relapse, median (IQR)**	**Relapse, median (IQR)**	**p-value**	**q-value**	**No relapse, median (IQR)**	**Relapse, median (IQR)**	**p-value**	**q-value**
**CD28**	1.65 (0.31-10.15)	0.40 (0.14-8.56)	0.56	0.70	0.68 (0.12-14.75)	12.76 (1.47-40.32)	0.036	0.25
**CD40L**	0.13 (0.11-0.61)	0.40 (0.15-0.86)	0.19	0.73	0.23 (0.042-0.67)	0.088 (0.0-0.66)	0.38	0.91
**CD57**	42.08 (30.47-53.77)	33.00 (20.01-53.30)	0.45	0.76	42.47 (30.99-67.40)	60.43 (31.24-64.75)	0.62	0.91

**CD69**	9.26 (6.20-11.74)	10.4 (7.84-14.58)	0.51	0.70	9.09 (5.46-13.42)	10.95 (7.04-15.35)	0.44	0.91
**CD95**	6.63 (3.60-9.47)	11.59 (2.70-18.80)	0.33	0.70	2.77 (1.64-6.50)	8.91 (1.81-13.97)	0.20	0.68
**CD134**	1.21 (0.62-3.47)	2.39 (1.25-4.27)	0.24	0.70	0.73 (0.40-1.43)	1.01 (0.73-1.48)	0.62	0.91
**CD137**	1.82 (1.04-5.46)	4.53 (2.63-21.98)	0.078	0.73	1.84 (1.37-4.70)	2.97 (1.43-7.63)	0.69	0.91
**CD160**	0.45 (0.11-0.70)	0.52 (0.11-2.35)	0.40	0.025	0.37 (0.10-0.79)	0.64 (0.32-2.10)	0.15	0.65
**CTLA-4**	1.61 (0.76-2.59)	3.83 (2.66-4.14)	0.0012	0.95	0.91 (0.38-1.47)	5.17 (1.03-7.15)	0.0092	0.19
**GITR**	0.28 (0.11-0.72)	0.48 (0.053-0.99)	0.95	0.70	0.12 (0.0-0.37)	0.046 (0.0-0.19)	0.57	0.91

**GzmB**	65.87 (44.84-76.21)	50.00 (27.77-67.34)	0.20	0.80	50.68 (35.07-74.45)	64.03 (28.14-74.87)	0.94	0.94
**IFN-γ**	0.044 (0.0-0.13)	0.031 (0.0-0.82)	0.65	0.80	0.042 (0.0-0.17)	0.025 (0.0-0.12)	0.65	0.91
**IL-2**	0.034 (0.0-0.14)	0.087 (0.0-0.66)	0.69	0.70	0.017 (0.0-0.081)	0.021 (0.0-0.046)	0.89	0.94
**IL-10**	0.0 (0.0-0.027)	0.087 (0.0-0.44)	0.29	0.95	0.0 (0.0-0.091)	0.0 (0.0-0.15)	0.31	0.91
**Ki-67**	3.85 (0.86-10.84)	2.60 (0.98-9.60)	0.95	0.70	0.57 (0.14-1.22)	0.49 (0.26-3.09)	0.81	0.94
**KLRG1**	21.32 (3.64-27.60)	12.40 (9.65-19.21)	0.33	0.70	15.47 (7.08-36.57)	12.78 (1.73-31.30)	0.48	0.91
**LAG-3**	0.18 (0.037-0.56)	0.029 (0.0-0.41)	0.32	0.95	0.0 (0.0-0.091)	0.021 (0.0-0.15)	0.89	0.94

**PD-1**	5.60 (1.31-7.84)	4.4 (3.32-7.73)	0.95	0.70	2.38 (1.35-3.66)	2.29 (1.59-5.37)	0.77	0.94
**TCF-1**	0.16 (0.065-1.40)	0.74 (0.25-5.06)	0.30	0.73	0.38 (0.11-1.12)	1.49 (0.37-3.82)	0.070	0.37
**TIGIT**	4.44 (0.99-7.82)	4.25 (2.72-7.77)	0.43	0.80	2.95 (2.05-3.87)	4.92 (0.90-7.70)	0.55	0.91
**TIM-3**	3.55 (0.50-7.48)	2.39 (0.60-5.43)	0.62	0.70	2.50 (0.67-8.07)	0.13 (0.0-3.50)	0.036	0.25

Abbreviations: IQR – interquartile range, CLTA-4 - cytotoxic T lymphocyte-associated protein 4, GITR - glucocorticoid-induced tumor necrosis factor receptor-related protein, IFNG – interferon gamma, GzmB – granzyme B, IL - interleukin, KLRG1 - killer cell lectin-like receptor subfamily G member 1, LAG-3 - lymphocyte activation gene 3, PD-1 - programmed cell death protein 1, TCF-1 - T cell factor 1, TIGIT - T cell immunoreceptor with Ig and ITIM domains, TIM-3 - T cell immunoglobulin and mucin domain-containing protein 3.

Supplementary References

1 Hall, V. G. *et al.* Humoral and cellular immune response and safety of two-dose SARS-CoV-2 mRNA-1273 vaccine in solid organ transplant recipients. *Am J Transplant* **21**, 3980-3989, doi:10.1111/ajt.16766 (2021).

2 Hall, V. G. *et al.* Delayed-interval BNT162b2 mRNA COVID-19 vaccination enhances humoral immunity and induces robust T cell responses. *Nat Immunol* **23**, 380-385, doi:10.1038/s41590-021-01126-6 (2022).

3 Diz, A. P., Carvajal-Rodriguez, A. & Skibinski, D. O. Multiple hypothesis testing in proteomics: a strategy for experimental work. *Mol Cell Proteomics* **10**, M110 004374, doi:10.1074/mcp.M110.004374 (2011).
